# Auxin Modulated Initiation of Lateral Roots Is Linked to Pericycle Cell Length in Maize

**DOI:** 10.3389/fpls.2019.00011

**Published:** 2019-01-24

**Authors:** M. Victoria Alarcón, Julio Salguero, Pedro G. Lloret

**Affiliations:** ^1^Departamento de Hortofruticultura, Instituto de Investigaciones Agrarias “La Orden-Valdesequera”, CICYTEX, Junta de Extremadura, Badajoz, Spain; ^2^Departamento de Anatomía, Biología Celular y Zoología, Facultad de Ciencias, Universidad de Extremadura, Badajoz, Spain; ^3^Departamento de Biología Vegetal, Ecología y Ciencias de la Tierra, Universidad de Extremadura, Badajoz, Spain

**Keywords:** lateral root development, auxin action, root elongation, pericycle, maize, *Zea mays*, cell growth

## Abstract

Auxin is essential for the regulation of root system architecture by controlling primary root elongation and lateral root (LR) formation. Exogenous auxin has been reported to inhibit primary root elongation and promote the formation of LRs. In this study, LR formation in the *Zea mays* primary root was quantitatively evaluated after exogenous auxin treatment by comparing the effects of auxin on two selected zones elongated either before or after auxin application. We determined two main variables in both zones: the LR density per unit of root length (LRD), and the mean phloem pericycle cell length. The total number of phloem pericycle cells (PPCs) per unit of root length was then calculated. Considering that each LR primordium is initiated from four founder cells (FCs), the percentage of PPCs (%PPC) that behave as FCs in a specific root zone was estimated by dividing the number of pericycle cells by four times the LRD. This index was utilized to describe LR initiation. Root zones elongated in the presence of a synthetic auxin (1-naphthalene acetic acid, NAA) at low concentrations (0.01 μM) showed reduced cell length and increased LRD. However, a high concentration of NAA (0.1 μM) strongly reduced both cell length and LRD. In contrast, both low and high levels of NAA stimulated LRD in zones elongated before auxin application. Analysis of the percentage of FCs in the phloem pericycle in zones elongated in the presence or absence of NAA showed that low concentrations of NAA increased the %PFC, indicating that LR initiation is promoted at new sites; however, high concentrations of NAA elicited a considerable reduction in this variable in zones developed in the presence of auxin. As these zones are composed of short pericycle cells, we propose that short pericycle cells are incapable to participate in LR primordium initiation and that auxin modulated initiation of LRs is linked to pericycle cell length.

## Introduction

Auxin is considered a key regulator of root growth, gravitropism, and lateral root (LR) formation (Muday and Haworth, 1994). Exogenous auxin treatment has typically resulted in inhibited root growth rate (Zeadan and Macleod, 1984; Hurren et al., 1988; Lloret and Pulgarín, 1992; Gaspar et al., 2003). Nevertheless, in *Arabidopsis* roots, it has been demonstrated that low concentrations of auxin can substantially stimulate primary root elongation (Evans et al., 1994). Auxin action on root development is not only a question of concentration but also of its polar translocation (Muday and DeLong, 2001). The auxin indole-3-acetic acid (IAA) is predominantly synthesized in shoots and transported basipetally from the apex to the base of the shoot (Lomax et al., 1995; Muday and DeLong, 2001; Casson and Lindsey, 2003). Once in the root, IAA moves acropetally toward the root apex through the central cylinder (Mitchell and Davies, 1975; Muday and DeLong, 2001) and basipetally from the root apex toward the elongation zone through the outer root tissues (Ohwaki and Tsurumi, 1976; Tsurumi and Ohwaki, 1978; Rashotte et al., 2000; Muday and DeLong, 2001; Ruzicka et al., 2007). Acropetal transport of IAA in the root is involved in the regulation of LR formation in *Arabidopsis* (Reed et al., 1998). Because basipetal IAA transport controls elongation of epidermal cells, it has been implicated in the regulation of gravitropism (Rashotte et al., 2001). As application of the auxin transport inhibitor naphthylphthalamic acid (NPA) to the tip of *Arabidopsis* roots inhibits both basipetal auxin transport and root elongation (Rashotte et al., 2000), it is likely that primary root elongation would also be controlled by the basipetal auxin transport mechanism. In some *Arabidopsis* mutants, it has been demonstrated that a reduced growth rate in primary roots is related, at least in part, to reduced elongation of individual cells (Hauser et al., 1995).

Auxin also regulates root system architecture by promoting the acquisition of founder cell (FC) identity in pericycle cells (Dubrovsky et al., 2008), and by stimulating LR development (Laskowski et al., 1995; Casimiro et al., 2003). Nevertheless, it has been reported that auxin loses its LR-promoting effect in newly formed regions of the *Arabidopsis* primary root growing at lower rate (Ivanchenko et al., 2010), suggesting that LR formation may be linked to cell length. Interestingly, regulation of initiation and subsequent development of LRs could be differentially controlled by basipetal (Casimiro et al., 2001) and acropetal auxin polar transport, respectively (Reed et al., 1998).

Lateral root initiation in maize begins with transversal divisions of pericycle cells associated with phloem poles, when two adjacent cells opposite the phloem undergo two almost simultaneous oblique asymmetrical divisions and later more transversal and periclinal divisions (Casero et al., 1995). In maize, periclinal divisions related to LR initiation occur 21–24 mm behind the tip (Casero et al., 1995). In most species, initiating LR primordia are located in the maturation zone (Lloret and Casero, 2002); consequently, the pericycle cells involved in this process are usually considered to be differenciated at the moment of LR initiation (Alarcón et al., 2016).

Several methods have been used to analyze LR development. The absolute number of LRs is not a good parameter of LR formation because it is strongly dependent on parent root length (Lloret and Pulgarín, 1992). Consequently, it evaluates not only LR formation but also main root elongation. The lateral root density (LRD) method determines the number of LRs per unit length of parent root. Currently, there is a trend to consider both emerged LRs and LR primordia, and to analyze exclusively the parent root zone that bears LRs and/or LR primordia (Lloret et al., 1988; Pulgarín et al., 1988; Dubrovsky et al., 2009). This is a better index of LR formation because it avoids distortions caused by the mother root zones where no LR initiation occurs. Another advantage of the LRD index is that it allows the comparison of LR formation in primary roots with different elongation rates. The recent implementation of the method to analyze LR formation through the determination of the “lateral root initiation index” (I_LRI_) represents a new and significant advance (Dubrovsky et al., 2009). This index describes the number of LR initiation anlages occurring along a root portion corresponding to 100 cortical cells in a file. The main advantage of using this index to describe LR initiation is that it offers a more integrative and cellular perspective, since it considers both the growth and rate of cell formation.

In this work, we show that exogenous auxin treatment of maize primary roots has a dual effect on LR formation, i.e., low doses promote the initiation of new primordia, whereas high doses inhibit this process. Therefore, the concept of auxin as a phytohormone which stimulates LR initiation should be re-evaluated, as was the role of ethylene as a general root growth inhibitor (Pierik et al., 2006). To explain the contrasting effects of auxin treatment on LR initiation, we propose that auxin modulated initiation of LRs is linked to pericycle FC length.

## Materials and Methods

### Plant Material and Growth Conditions

**FIGURE 1:**
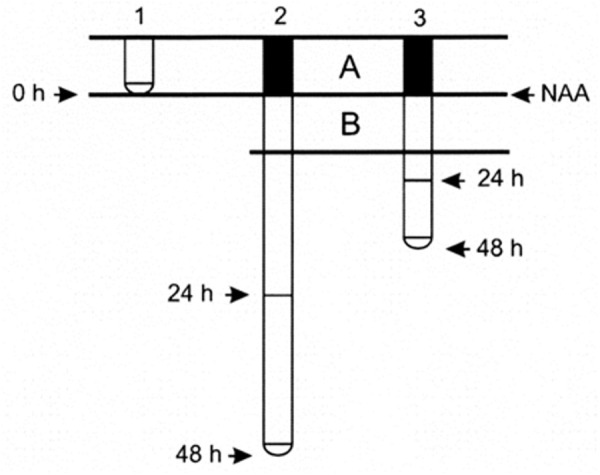
Formation of two 2-cm long consecutive zones in the maize primary root after NAA application. At time 0, treatment was performed by applying NAA to the growth medium (1). Zone A (filled) is elongated before auxin application, hence cell elongation occurred without auxin influence. In contrast, zone B (unfilled) elongated in the presence of auxin. Subsequently, the root grows and lateral roots develop in both zones under the influence of exogenous auxin. Note that after 48 h, root elongation was greater in control (2) than in auxin-treated roots (3).

Seeds of the *Zea mays* L. hybrid, DK 626, were sterilized by immersion in ethanol for 5 min. They were then washed three times and soaked in distilled water with aeration at 30°C. After 24 h, the seedlings, with radicles approximately 1 mm long, were placed vertically in holders made of Styrofoam disks, transferred into boxes, and placed in a humid environment, in the dark, at 30°C. They were kept in these boxes for 24 h until the roots reached a length of 20 ± 5 mm. Disks with 10 selected seedlings of equal root length were placed in bottles containing 1.5 L aerated growth solution and grown at 30°C in the dark. As Ca^2+^ and K^+^ are required for proper development of roots growing in hydroponic medium (Lee et al., 1983; Hasenstein and Evans, 1986; Walker et al., 1998), the growth medium consisted of a buffered solution of 1 mM HEPES (4-(2-hydroxyethyl)-1-piperazineethanesulfonic acid) supplemented with 1 mM Ca_2_Cl and 10 mM KCl at pH 6.0. HEPES was used as a buffering agent because it is highly soluble and chemically and enzymatically stable, in addition to having other advantages. The experimental conditions used in this study have been shown to be optimal for the growth of maize roots (Alarcón et al., 2009). After acclimation for 24 h, when the roots were 80 mm long, the synthetic auxin 1-naphthalene acetic acid (NAA) was added to the solution and the treatment was maintained for 48 h. Root length was measured with a ruler (accuracy ± 1 mm) before NAA application, and after 24 and 48 h of treatment. After NAA application, two 2-cm long consecutive root zones could be distinguished, that we called zones A and B (Figure 1). The proximal A zone elongated before auxin application, indicating that cell elongation occurred in the absence of the exogenous hormone. In contrast, the distal B zone grew in the presence of exogenous auxin. However, LR development occurred under exogenous auxin influence in both zones.

### Chemicals

Concentrated solutions of NAA were prepared in 1 mM HEPES buffer. The volumes added to the growth medium were less than 0.1% of the total volume. All the chemicals were purchased from Sigma, except CaCl_2_ and KCl (Merck).

### Determination of Lateral Root Density in Selected Root Zones

To allow the development of the LR primordia until they were clearly identifiable under a dissecting microscope to facilitate quantification, roots were cultivated in growth medium for 48 h. The primary root length was then measured and zones A and B were selected. The root zones were fixed in FAA fixative for 48 h. FAA is a mixture of 50% ethanol, 36% formaldehyde, and 100% acetic acid (91:6:3, v/v). The roots were subsequently transferred to 70% ethanol. LRs were quantified by observation under a dissecting microscope. The positions of the LRs were also recorded. The number of LRs that were formed during the experimental period was calculated as well as LRD in zones A and B. Both variables were determined as the number of LRs plus LR primordia per cm.

### Determination of Cell Length and Parameters of LR Formation

To measure cell length, 5-mm long central segments of zones A and B were selected. These segments were embedded in paraffin and cut into 8-μm serial longitudinal sections. Cell length was determined for at least five root segments for each treatment, measuring 12 cells per segment.

The calculation of the percentage of phloem pericycle cells (PPCs) that behave as FCs requires the previous determination of two variables in zones A and B, namely, LRD and PPC length. To estimate this percentage, each LR primordium is assumed to originate from four phloem pericycle FCs, since LR primordium initiation in maize has been described as involving asymmetrical transverse divisions of a pair of adjacent pericycle FCs in each file (Casero et al., 1995), and that each phloem pole in maize is surrounded by two phloem pericycle cell files. The new index for LR formation that we propose is based on the percentage of PPCs (%PPC) that behave as FCs. The percentage of PPC that become FCs was estimated by dividing the total number of FCs that gave rise to the LRs in a specific zone (equal to the number of LRs multiplied by four cells) by the total number of PPCs present within the same zone and by multiplying this ratio by 100. The results of %PPC are presented as the mean ± SD of at least five root segments per treatment.

Another index of LR formation (I_LRI_) was calculated as established by Dubrovsky et al. (2009) for *Arabidopsis* roots. This index was defined to analyze the number of LRs and/or LRPs that developed in a primary root segment containing 100 cortical cells. This index was also calculated in at least five root segments per zone, and per treatment.

The relative rate of PPC cell production could be linked to LR formation. This rate of cell production (RCP) (Ivanov and Dubrovsky, 1997) can be calculated for a specific time by dividing the elongation velocity of the primary root by the average cell length of the cell type of interest. We calculated the RCP as the number of PPCs produced per meristem file per unit of time. At least five root segments were used per experimental group.

### Measurements and Statistical Analysis

The values obtained for root elongation and LRD are represented as the mean ± SD of 10 roots. The other variables had a sample size of at least five root segments per experimental group. Each experiment was repeated at least twice. Comparison of multiple means was performed using one-way ANOVA and Tukey’s test (SPSS 13.0). Differences of means were considered significant at *P* < 0.05.

## Results

### Exogenous 1-Naphthalene Acid (NAA) Application Increases LRD

**FIGURE 2:**
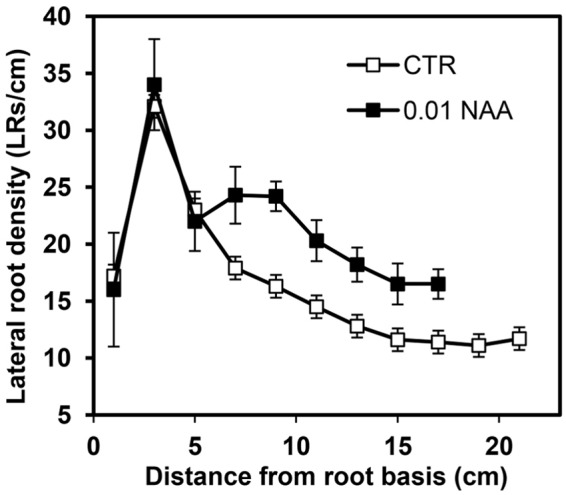
Effect of 0.01 μM NAA treatment on lateral root density (LRD) in the maize primary root. LRD was measured in 2-cm-long root segments from the base to the root tip. Auxin was applied when the roots were 8 cm long and LR formation was followed during a subsequent 48 h period. Note the inhibitory effect of NAA on root elongation and the stimulating effect on LRD in the youngest root zones analyzed. Values represent mean ± SD (*n* = 10).

The LR distribution pattern along the primary root of maize is shown in Figure 2. The number of LRs plus LR primordia per cm was determined in 2-cm long control and 0.01 μM NAA-treated root segments. The distribution of the LRs in control roots showed a characteristic pattern with several different zones. In the basal-most parent root segment, the density was relatively low (15–17 LRs/cm). In the segment between 2 and 4 cm from the base, the LRD then increased until the zone of highest density (30–35 LRs/cm), and then decreased sharply to 15 LRs/cm in the segment located 6–8 cm from the root base. From this point toward the apex, the LRD declined slowly and then stabilized at a value close to 12 LRs/cm. LR primordia were not detected in the zone adjacent to the root apex.

The application of 0.01 μM NAA stimulated the formation of LRs in the primary root of maize. This stimulation was observed as a wide extension of the parent root, mainly affecting the more distal root segments (Figure 2); in the youngest root segments grown after NAA treatment, significant LRD stimulation (treated: 24.3 ± 3.0 LRs/cm vs. control: 15.9 ± 2.5 LRs/cm) was observed. Promotion of LR formation also occurred in new segments formed after beginning of treatment. In contrast, more proximal root zones were apparently insensitive to auxin treatment (Figure 2).

In addition, application of 0.01 μM NAA inhibited primary root elongation by 30% and reduced the number of segments along the root where LRs normally form. Consequently, a relationship between the parent root growth and LR formation was analyzed in more details.

### Relationship Between Primary Root Elongation and Lateral Root Formation

**FIGURE 3:**
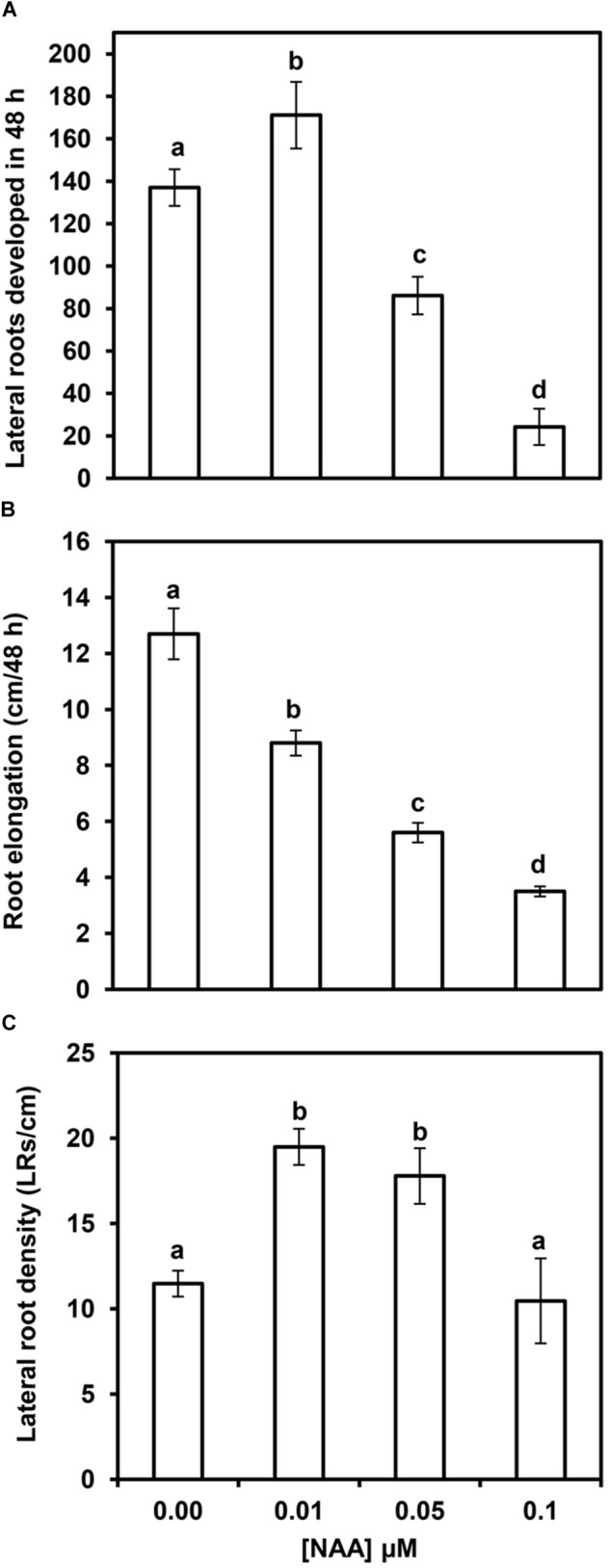
Effects of several NAA concentrations on lateral root (LR) formation and primary root elongation within 48 h after NAA application. Number of LRs plus LR primordia **(A)**, primary root elongation **(B)**, and average LRD **(C)** in the formed segments of primary maize roots. Values are mean ± SD (*n* = 10). Different letters indicate significant differences at *P* < 0.05 (ANOVA and Tukey’s test).

Maize primary root elongation was inhibited by NAA treatment in a concentration-dependent manner, with a consequent reduction of the area where LRs can develop (Table 1). A low NAA concentration (0.01 μM) marginally inhibited primary root elongation, but increased the total number of LRs after 48 h of treatment (Figures 3A,B). However, higher NAA concentrations elicited a large reduction in the absolute number of LRs (Figure 3A), as well as a strong concomitant reduction in primary root elongation. Therefore, the inhibition of LR formation could be due, at least in part, to the reduction in primary root elongation. Concentrations of NAA in the 0.05–0.1 μM range resulted in stronger inhibition of root elongation (up to 75%), and 0.5 μM NAA completely inhibited primary root elongation (data not shown). When primary root elongation is inhibited, LR primordia have limited physical space to develop, and LRD (number of LRs and/or LR primordia per unit length) may therefore be a better index to evaluate LR formation than the absolute number of LRs. Figure 3C shows that 0.01 and 0.05 μM NAA significantly increased the average LRD. However, the stimulatory effect was lost with application of higher doses of NAA. This suggests that high concentrations of auxin, which resulted in strongly reduced primary root elongation, inhibit LR formation.

**Table 1:** Concentration-dependent effects of NAA on cell length and root elongation.

Zone	Groups	Root elongation (mm/48 h)	ECL (μm)	CCL (μm)
A	0.00 NAA	129.3 ± 9.1 (100)a	152.8 ± 6.3 (100)a	191.9 ± 14.1 (100)a,b
A	0.01 NAA	–	144.4 ± 17.5 (95)a	182.7 ± 9.5 (95)a,b
A	0.05 NAA	–	150.6 ± 18.9 (99)a	168.6 ± 11.0 (88)b
A	0.10 NAA	–	151.6 ± 21.3 (99)a	204.4 ± 15.5 (107)a
B	0.00 NAA	128.4 ± 17.3 (100)a	154.8 ± 20.5 (100)a	195.6 ± 13.1 (100)a
B	0.01 NAA	97.4 ± 17.0 (76)b	121.2 ± 10.5 (78)b	167.0 ± 16.0 (85)b
B	0.05 NAA	50.7 ± 9.3 (39)c	60.6 ± 11.4 (39)c	91.8 ± 10.5 (47)c
B	0.10 NAA	31.8 ± 3.5 (25)d	48.5 ± 2.5 (31)c	65.2 ± 11.3 (33)d

*The mean cell length of the epidermis (ECL) and cortex (CCL) in zones A and B under different experimental conditions are indicated. Different letters indicate significant differences compared to control roots (ANOVA, *P* < 0.05). Cell length values are the mean ± SD of at least five roots (measuring 12 cells per root). The sample size for the root elongation rate was 18 roots. Values in parentheses indicate the percentage compared to untreated roots in the same zone. Zone A developed before NAA treatment, and zone B after NAA treatment.*

Moreover, strong inhibition of primary root elongation could result in segments located in the same position in both control and treated roots not forming at the same time (Figure 1). As segments at the same position would not be homologous, comparison of LRD between control and treated roots becomes difficult, and even meaningless in extreme cases. Therefore, it is appropriate, as far as possible, to compare root segments of the same age and similar relative location along the primary root (homologous segments).

### Root Zones Formed Before and After NAA Treatment

**FIGURE 4:**
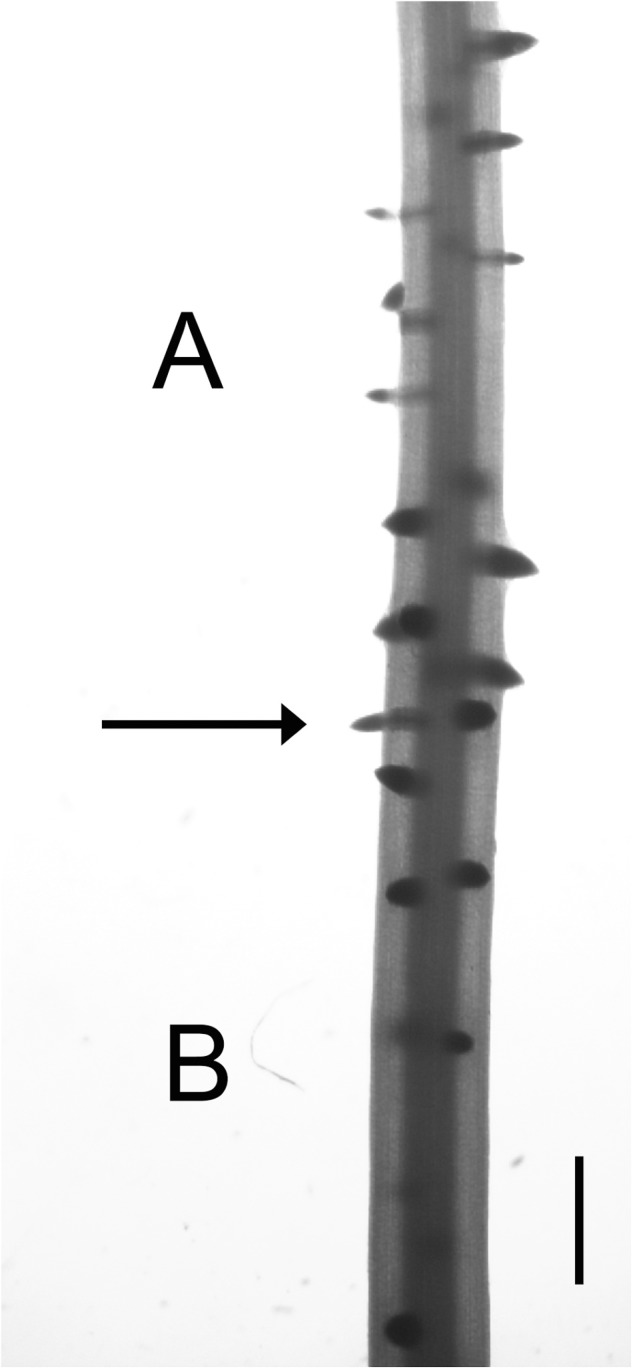
The junction between zones **A** and **B** in 0.1 μM NAA-treated maize roots. Micrograph of a maize root fragment at the junction between zones **A** and **B** (arrow). Note that zone **A** zone is thinner and shows a high LRD with numerous emerged lateral roots (LRs). In contrast, zone **B** is thicker, with relatively few un-emerged LRs. Scale bar = 1 mm.

At the beginning of NAA treatment, there were two homologous zones in control and treated roots which were formed. We called these zone A and zone B (Figure 1), and they exhibit several clear differentiating features. The main difference between the two zones was that the A zone elongated in the absence of exogenous auxin, but LR development occurred in the presence of NAA; as meanwhile, the B zone elongated and initiated LR primordia in the presence of auxin. Corresponding zone in untreated roots was defined as a root portion formed during the same time. Additionally, these two zones showed different thickness, LRD, and stages of LR primordium development (Figure 4).

Auxin application elicits a progressive increase in the thickness of zone A (Figure 4), without apparently affecting LR primordium initiation or development (Figure 4). In contrast, LR development appeared to be altered in the B zone (Figure 4). The existence of these two segments that developed in a short period, but under different physiological conditions, allows the separation of the effects of auxin on root elongation and LR formation.

### NAA Application Elicits Different Effects on LRD in Zones A and B

The obtained LRD values for untreated primary roots for zones A and B were 18.8 ± 2.9 and 17.6 ± 1.8, respectively (Table 2). These results did not differ significantly (*P* > 0.05, ANOVA and Tukey’s test for unplanned comparisons). The application of NAA at 0.01, 0.05, and 0.1 μM concentrations significantly increased LRD in zone A. This variable increased from 18.8 ± 2.9 in untreated roots to an average of approximately 25 LRs per cm in NAA-treated roots. No significant differences in LRD were found between different NAA concentrations, showing that the stimulatory effect seen in zone A was independent of NAA concentration (Table 2). In contrast, the effect of NAA on LR development in zone B was concentration-dependent. As in zone A, application of 0.01 μM NAA resulted in a significant increase in LRD. However, with 0.05 μM NAA, LRD stimulation was lost, and was reduced to values lower than observed for control roots with the highest NAA doses (Table 2).

**Table 2:** Lateral root formation in maize roots under different exogenous auxin treatments.

Zone	Groups	PPCL	LRD	%FC	I_LRI_	RCP
A	0.00 NAA	264.3 ± 22.8a	18.8 ± 2.9a	6.5 ± 0.5a	36.0 ± 5.0a	7.8 ± 1.3a
A	0.01 NAA	279.0 ± 18.1a	25.5 ± 2.8b	10.4 ± 1.2b	50.9 ± 5.2b	6.9 ± 1.6a
A	0.05 NAA	268.8 ± 12.5a	25.9 ± 2.5b	9.6 ± 1.0b	45.2 ± 4.4b	7.7 ± 0.5a
A	0.10 NAA	277.2 ± 32.4a	24.8 ± 2.6b	9.1 ± 1.6b	50.2 ± 7.3b	7.8 ± 1.4a
B	0.00 NAA	265.8 ± 12.8a	17.6 ± 1.8a	6.1 ± 0.9a	33.9 ± 4.7a	9.6 ± 0.4a
B	0.01 NAA	248.1 ± 40.7a	24.9 ± 1.4b	8.2 ± 1.2b	41.6 ± 4.8b	7.2 ± 1.7b
B	0.05 NAA	139.4 ± 23.2b	21.5 ± 3.3c	4.3 ± 0.6c	21.4 ± 1.8c	6.2 ± 0.7b
B	0.10 NAA	110.1 ± 17.4b	10.6 ± 2.6d	1.7 ± 0.3d	7.5 ± 1.4d	6.1 ± 0.9b

*The data correspond to zones A and B of the primary root formed, respectively, before or after different NAA treatments (groups). Phloem pericycle cell length in μm (PPCL), mean number of lateral roots per cm of parent root (LRD), percentage of founder cells (%FC), index of lateral root initiation (I_LRI_) as defined by Dubrovsky et al. (2009), and root production rate per cell file per hour for phloem pericycle cells (RCP) are presented. The figures correspond to mean values ± SD. Different letters indicate statistically significant mean differences within each column and zone (one-way ANOVA, Tukey’s test, *P* < 0.05). Values for pericycle cell length correspond to five roots, measuring at least 15 cells per root. The LRD was calculated from 10 roots. The percentage of FCs was calculated based on known values of LRD and PPCL, and assuming that the maize root has 15 poles of phloem, each of them with two columns of pericycle cells, and that each LR is initiated from four PPCLs.*

### Reduction of Epidermal and Cortical Cell Length by NAA Treatments

We measured epidermal and cortical cell length in zones A and B of both control and NAA-treated roots. The majority of cells in zone A had finished elongating at the onset of auxin treatment. Therefore, we expected that NAA application would not affect cell length in zone A. Indeed, our results showed that the length of epidermal cells was not reduced in this zone with any NAA dose used (Table 1); moreover, cortical cell length in zone A also showed a tendency to maintain cell length. In relation to cortical cell length, the tendency was also to maintain similar values in both treated and control roots. The minimum length was recorded in roots treated with 0.05 μm NAA (12% reduced vs. control roots). However, this reduction in cell length was not statistically significant (Table 1).

In zone B, inhibition of root elongation by NAA was always accompanied by a reduction in the length of epidermal and cortical cells (Table 1). This reduction was progressive and always statistically significant, except for the epidermal cells of the group treated with 0.1 μM NAA, where cell length was 48.1 μm, likely near the lower limit for non-elongated cells (Table 1).

### Reduction of Pericycle Cell Length by NAA Results in Decreased LRD

To study a possible relationship between pericycle cell length and LR formation, both parameters were analyzed comparatively in roots treated with several concentrations of NAA (Table 2). The mean PPC length in zone A of control roots was 264.3 ± 22.8 μm. In a given root segment, the number of PPCs in a cell file can be calculated by dividing the total length of the segment by the mean length of PPCs. In our sample, the mean number of phloem poles was 15 and the number of PPC cell files at each phloem pole was calculated as two. Consequently, the total number of PPCs in a specific root segment can be estimated by multiplying by 30 (2 PPC cell files per pole and 15 phloem poles) the average number of cells in a pericycle cell file along the entire length of the segment.

As we consider that each LR primordium originated from four FCs per phloem pole, and LRD was 18.8 ± 2.9 in zone A of control roots, the percentage of FCs out of total number of PPC is equal to 6.5 ± 0.5 (Table 2). Application of 0.01, 0.05, or 0.1 μM NAA did not significantly affect the length of pericycle cells in zone A, but the LRD was significantly increased (Figure 3C). Hence, the percentage of FCs increased from 6.5 to 10.4, 9.6, and 9.1, respectively, after NAA treatment (Table 2). A similar increase in LR formation was observed when the lateral root initiation index (I_LRI_) was calculated according to Dubrovsky et al. (2009).

In zone B of untreated roots, the values for PPC length, LRD, percentage of FCs, and I_LRI_ (265.8 μm, 17.6 LRs/cm, 6.1%, and 33.8, respectively) were similar to those found in zone A (Table 2). Application of 0.01 μM NAA promoted a significant increase in LRD (24.9 LRs/cm) in zone B, which was accompanied by a parallel increase in the percentage of FCs (8.2%) and I_LRI_ (41.6%) compared with control roots (Table 2). However, treatments with higher concentrations of NAA significantly reduced the mean PPC length, LRD, percentage of FCs, and I_LRI_ (Table 2). Taken together, these data clearly show that the inhibition of PPC elongation by high NAA concentrations resulted in a reduced rate of LR formation.

We also determined the RCP values to establish whether there was a reduction in pericycle cell production in roots subjected to treatments with high concentrations of auxin. It was possible that this reduction could be linked to a decrease in the rate of LR primordium production. In control roots, this variable showed values of 7.8 ± 1.3 and 9.6 ± 0.4 cells per row per hour for zones A and B, respectively, when they formed (Table 2). This variable decreased in treated roots until reaching a minimum value of 6.1 ± 09 PPCs per cell file per hour in zone B. Consequently, there was a reduction of approximately 35% in the RCP. However, the apical meristem of the root never ceased producing new cells.

## Discussion

### Competence Period for LR Initiation

Studies in different species have tended to confirm that pericycle cells respond only to factors that trigger LR initiation during a short period of time, called the competence period or developmental window (Abadía-Fenoll et al., 1986; Dubrovsky et al., 2006). The existence of a competence period results in LR initiation following an essentially acropetal sequence, with new LR primordia initiating distally to those formed previously (Lloret and Casero, 2002). According to Dubrovsky and Rost (2012), at least two events must occur in *Arabidopsis thaliana* roots before the first cell divisions of LR primordium organogenesis occur, namely, priming and specification of pericycle FCs. Priming can be considered as cell commitment to LR formation. In most higher plant species, this phenomenon occurs in the transition zone between the root apical meristem and the elongation zone (Dubrovsky and Rost, 2012). Specification consists of pericycle FCs acquiring a developmental fate different from other pericycle cells, and participating in LR primordium initiation (Dubrovsky and Rost, 2012). Local accumulation of free auxin has been shown to be a prerequisite for FC specification (Dubrovsky et al., 2008). Priming and specification can occur within a time interval shorter than a cell cycle (Dubrovsky and Rost, 2012). Consequently, these two processes should be concluded when pericycle cells eventually reach the end of the elongation zone, explaining the narrow developmental window for LR initiation.

Our results presented in Figure 2 support the hypothesis of the existence of a short competence period for LR initiation. Indeed, only the youngest root fragments increase their LRD in response to treatment with 0.01 μm NAA. Stimulation of LR formation by auxin in newly formed root zones is expected as this hormone is considered the main regulator of LR formation (Lloret and Pulgarín, 1992; Hobbie, 1998; Lloret and Casero, 2002), and numerous young pericycle cells capable of becoming FCs exist in this region (Dubrovsky et al., 2006). In the preformed regions close to the root apical meristem, we also found that NAA treatment stimulated LR formation. This suggests that pericycle cells that are primed, but not specified, will eventually develop LRs as a result of the treatment. In this case and under normal conditions, the possible regions for LR formation would be more abundant than regions where LR primordia will eventually develop.

### Pericycle Cell Length and Auxin Response During LR Formation

It is well-known that exogenous auxin treatment normally promotes LR formation in several plant species (Zeadan and Macleod, 1984; Hurren et al., 1988; Lloret and Pulgarín, 1992; Zhang and Hasenstein, 1999; Nieuwland et al., 2009). Overall, the high auxin concentrations used in these experiments also inhibited primary root elongation, posing the question as to whether both effects of auxin on root system development are related. Recent evidence suggests complex effects of auxin on LR initiation in *A. thaliana* roots showing reduced cell elongation (Ivanchenko et al., 2010). The above mentioned study demonstrated that when the primary root elongation rate is reduced, the promotion of LR formation by auxin diminishes with increasing concentrations of this phytohormone. Our results in *Z. mays* agree entirely with those obtained in *A. thaliana* roots. The suggested connection between inhibition of primary root elongation and reduced LR formation would be mediated by a decrease in the sensitivity of pericycle cells to auxin under conditions of reduced growth (Ivanchenko et al., 2010).

We propose an alternative hypothesis to explain the same phenomenon in maize roots. The loss of the ability of auxin to stimulate LR formation when applied at high concentration may be due to the substantial reduction in cell length occurring in the pericycle of maize under conditions of reduced growth. Notably, in the study by Ivanchenko et al. (2010), a maximum inhibition of approximately 8% in cortical cell length was achieved, whereas in our experiments we observed a reduction in length of nearly 60% (Table 2). Our hypothesis does not exclude that other factors apart from cell length may be linked to the mechanism that controls LR formation under conditions of reduced growth of the primary root. However, variations in the length of pericycle FCs fully and reasonably justify the behavior of LR initiation observed in our experiments.

Our analysis of LRD in zones A and B of the maize root is especially important. Zone A corresponded to the apical-most 2 cm of primary root length when NAA treatment began and, consequently, had a pericycle too young to show advanced stages of LR development. Zone B was generated by the apical meristem during NAA treatment and new LRs were formed during the same time and under the same treatment. Nevertheless, the elongation process was different between the two root zones. In our experiment, zone A cells located proximally to the elongation zone grew without the influence of auxin. In contrast, in the B zone, the root elongation process occurred in the presence of auxin. Consequently, this experimental system can discriminate whether an effect on LR formation is independent or dependent of root elongation.

In our experiments, roots treated with 0.01 and 0.05 μM NAA showed increased LRD, in both the A and B zones. Nevertheless, strong NAA doses (0.1 μM) increased LRD in zone A, but not in zone B. This indicates that the promoting effect of auxin on LR formation is lost in the latter zone with 0.1 μM auxin treatment. The situation in zone B contrasted with the stimulation of LRD in zone A after the same auxin treatment. The most likely explanation for this phenomenon is that the high auxin concentration used in this experiment interacts with another factor to elicit a differential response in zones A and B.

Maize LRs originate from pericycle FCs located opposite the phloem poles of the parent root (Casero et al., 1995). The initiation of LRs depends on a controlled sequence of cell divisions, some of which are asymmetrical (Casero et al., 1995). Studies in diverse organisms, from bacteria to vertebrates, indicate that cell cycle commitment to the G1 to S phase transition requires that cells grow to a critical size, which is an active mechanism to ensure cell size homeostasis (Dungrawala et al., 2012). Recent evidence in plants also suggests that cell size is one of the variables controlling cell cycle progression (Rioboo et al., 2009; Bush et al., 2015; Löfke et al., 2015; Jones et al., 2017). Cells must have a mechanism for coordinating their rates of cell growth and division. In budding yeast, growth to a “critical cell size” must be achieved before cells pass the check point known as START and transition from the G1 to the S phase (Dungrawala et al., 2012). The START point is equivalent to the restriction point in mammalian cells (Dungrawala et al., 2012). Therefore, it is possible that most pericycle cells in zone B in our study, had lost their ability to proliferate and launch LR primordium initiation as a result of their extremely short length after treatment with auxin 0.1 μM NAA. Conversely, it has been demonstrated that when the *Arabidopsis* root is bent there is a strict correlation between cell size, auxin transport, and stimulation of LR formation in the convex side of the curvature (Laskowski et al., 2008). In this system, a maximal level of auxin is established on the convex side of the curvature where LR primordia are initiated, whereas on the concave side of the curvature, where short pericycle cells are located, LR primordia do not develop.

As with other developmental processes, LR initiation requires that a series of asymmetrical cell divisions take place (Casero et al., 1995). Alterations in cell expansion could affect not only the ability of pericycle cells to divide, but also their ability to divide asymmetrically. During the segmentation of new colonies of the green alga *Volvox*, gonidial cells are produced after asymmetrical cell divisions. It has been demonstrated that the large size of the gonidial cells is the primary reason for differences in cell fate, morphologic characteristics, and cell division behavior compared to somatic cells (Kirk et al., 1993). In plants, the polarized expansion of the wall may be a mechanism that promotes asymmetries in cell division (Menke and Scheres, 2009). In addition, it has been proposed an inhibition of asymmetrical divisions in short pericycle cells during LR initiation (De Smet et al., 2008).

Moreover, it was recently shown that overexpression of the small peptide GLV6 disrupted the first asymmetric divisions required for the formation of a viable LR primordium, resulting in a reduced number of emerged LRs (Fernandez et al., 2015). It is well known that GLV peptides are also implicated in the regulation of cell elongation (Ghorbani et al., 2016). Consequently, pericycle cell elongation and LR initiation are likely to be interrelated processes.

The reduction in the density of LR primordia observed when NAA was applied at high concentrations could be related to a reduction in the rate of production of cells in the root apical meristem, or a consequence of the shortening of pericycle cell length. Indeed, cell became shorter even though NAA did not completely inhibit root elongation. Importantly, the RCP value for the PPC responsible for the initiation of LR primordia was maintained at a level of approximately six cells per cell file per hour (Table 2), apparently sufficient to promote a high density of LR primordia. However, the results presented here suggest that the specification process for LR initiation in PPCs is highly compromised when cell elongation is strongly reduced.

Therefore, pericycle cell length could play an important role in regulating the formation of LRs. For maize roots at least, LR initiation can no longer be interpreted only from an organismal perspective. In pericycle cells, in addition to regulatory effects that operate at the level of the whole plant, cell length appears to be a possible modulator in the control of LR initiation.

### Quantification of LR Formation

The quantification of LR formation is an important but somewhat complex topic (Dubrovsky et al., 2009). One difficulty to consider is that both already emerged LRs and endogenous LR primordia are not visible unless you clear the root or obtain histological sections. Another difficulty is that comparisons must frequently be established between roots of different ages and lengths that have been growing at very different growth rates. Hence, our results should be normalized to enable meaningful comparisons between root zones with homologous development (Figure 1).

To compensate for the influence of the primary root length on LR formation, two useful strategies have been developed to date. The first is to divide the number of LRs (independently of whether they are LR initiation events or already emerged roots) by the length of the region of the primary root containing both LRs and LR primordia (Lloret et al., 1988; Lloret and Pulgarín, 1992). A regularly used alternative option for this type of calculation has been to divide the length of the primary root into segments of fixed length and recount the number of LRs present in each segment (Hummon, 1962; Lloret et al., 1988; MacLeod, 1990; Varney et al., 1991; Lloret and Pulgarín, 1992). The second strategy consists of relating LR formation indices to the intervening cells between successive initiation events (Lloret et al., 1985; Dubrovsky et al., 2009). This protocol is useful because it adjusts the results for the differences in primary root growth rate. Both methods have advantages and disadvantages, but indices based on the latter type are the most appropriate from the point of view of developmental biology because they reveal whether the FCs were produced by the root apex at a regular rhythm.

However, not all FCs formation result in LR development, and then the longitudinal spacing pattern of LRs becomes more difficult to evaluate (Charlton, 1983; Barlow and Adam, 1988; Lloret and Casero, 2002). Recently, this issue was revisited in the model plant *A. thaliana* (Dubrovsky et al., 2006). In the same species, an index based on cortical cell length, called the lateral root initiation index (I_LRI_), has been applied to study LR distribution patterns (Dubrovsky et al., 2009). Here, we present a new method to evaluate LR formation that is relatively simple but highly effective in estimating LR formation, namely, the percentage of FCs.

The I_LRI_ proposed by Dubrovsky et al. (2009) defines root segments as a function of the length of fully elongated cortical cells. Nevertheless, LR initiation starts in pericycle FCs, and it may be more appropriate to relate the index to pericycle cells instead of cortical cells. Therefore, we directly calculated the percentage of FCs. Despite the differences, these indices produced similar estimations of LR formation under different experimental conditions (see Table 2). For example, the increase in the percentage of pericycle FCs compared to the control was 48% in roots treated with 0.05 μM NAA. This change is consistent with the reduced length of epidermal or cortical cells occurring under the same treatment (39 and 47%, see Table 1). In contrast, an increase of approximately 30% is observed if the index is calculated using cortical cell length (I_LRI_).

In summary, a new index based on the percentage of pericycle cells that produce LR primordia is presented in this report. This index provides a more accurate tool for the estimation of LR formation, as the measurements are made on pericycle FCs which initiate LR formation, and are the most directly implicated in this process. In addition, we compared this new index with a previous index based on cortical cell length and obtained similar results. The results of our study also showed that auxin modulated initiation of LRs is closely linked to pericycle cell length.

## Author Contributions

MA, JS, and PL conceived the study, contributed to methodology, wrote the original draft, and acquired the funding.

## Conflict of Interest Statement

The authors declare that the research was conducted in the absence of any commercial or financial relationships that could be construed as a potential conflict of interest.
